# Multiple Hypotheses Testing Procedures in Clinical Trials and Genomic Studies

**DOI:** 10.3389/fpubh.2013.00063

**Published:** 2013-12-09

**Authors:** Qing Pan

**Affiliations:** ^1^Department of Statistics, The George Washington University, Washington, DC, USA

**Keywords:** false discovery rate, family wise error rate, global test, multiple hypotheses testing, resampling method, stepwise procedure

## Abstract

We review and compare multiple hypothesis testing procedures used in clinical trials and those in genomic studies. Clinical trials often employ global tests, which draw an overall conclusion for all the hypotheses, such as SUM test, Two-Step test, Approximate Likelihood Ratio test (ALRT), Intersection-Union Test (IUT), and MAX test. The SUM and Two-Step tests are most powerful under homogeneous treatment effects, while the ALRT and MAX test are robust in cases with non-homogeneous treatment effects. Furthermore, the ALRT is robust to unequal sample sizes in testing different hypotheses. In genomic studies, stepwise procedures are used to draw marker-specific conclusions and control family wise error rate (FWER) or false discovery rate (FDR). FDR refers to the percent of false positives among all significant results and is preferred over FWER in screening high-dimensional genomic markers due to its interpretability. In cases where correlations between test statistics cannot be ignored, Westfall-Young resampling method generates the joint distribution of *P*-values under the null and maintains their correlation structure. Finally, the GWAS data from a clinical trial searching for SNPs associated with nephropathy among Type 1 diabetic patients are used to illustrate various procedures.

## 1 Introduction

When more than one hypotheses are tested at the same time, it is well known that the family wise type I error rate (FWER), that is, the probability of reporting at least one significant finding when the null hypotheses are true, will be inflated. Take *J* independent test statistics as an example. When each test controls its type I error rate at α level, the FWER is 1 − (1 − α)*^J^*. Table 1 lists the FWERs for different combinations of *J* and α. When *J* = 10 and α = 0.05, FWER goes up to 0.401. In cases of 100 or more simultaneous tests, it is almost sure to get false positive results.

**Table 1:** **FWER versus number of tests and the size of individual tests**.

α	*J*	*FWER*
0.01	2	0.020
0.01	5	0.049
0.01	10	0.096
0.01	100	0.634
0.01	1000	1.000
0.05	2	0.098
0.05	5	0.226
0.05	10	0.401
0.05	100	0.994
0.05	1000	1.000

Multiple hypotheses testing arises frequently both in clinical trials and in genomic studies. The different goals in these two settings result in different strategies. First, the hypotheses in clinical trials are often considered as a whole while those in genomic studies are more independent from each other. In clinic trials, multiple hypotheses are often considered jointly with a coherent theme. A few examples are given as follows. The symptoms of a complex disease often show up in different parts of the body or in different forms, such as different types of cancer. Multiple laboratory measurements monitor the underlying disease process, such as the viral loads and CD4 cell counts in HIV positive subjects. A treatment might have different responses in different patient sub-populations. On the other hand, multiple hypotheses in genomic studies arise because a large number of candidate markers are tested at the same time. Based on the number of tests carried out in the procedure, the multiple testing adjustment approaches can be grouped into global tests and stepwise procedures (1). Global tests summarize information from all endpoints/measurements/strata in one test statistic, while stepwise procedures carry out one test for each hypothesis. Therefore global tests are employed frequently in clinical trials while genomic studies almost always employ stepwise procedures. Second, the hypotheses in clinical trials are usually more specific with abundant prior information. In testing a specific treatment, with knowledge on the direction of the effects, directional tests with higher power can be employed. On the other hand, the genomic, epigenomic, transcriptomic, and proteomic network is much more complicated and often researchers screen for any signal, without knowing its direction or relationship to other markers. Third, the numbers of hypothesis in clinical trials are on a much smaller scale compared to the numbers in genomic studies – the numbers in clinical trials are usually less than ten, while the numbers of potential markers in genomic studies are sometimes over a million. In this manuscript, common procedures of multiple hypotheses adjustment in the two different settings are reviewed and compared.

The effects of interests are usually inferred from regression coefficients. In linear regression for normally distributed outcomes, the coefficient represents the difference in the outcome values between the groups being compared. In generalized linear models with logit link for binary outcomes, the coefficient equals the logarithm of the odds ratio of the outcome in the treatment group relative to the control group. In Cox proportional hazards models for partially censored failure time data, the exponentiated coefficient represents the hazards ratio. This review focuses on the choice of proper multiple testing adjustment method after the estimation procedures. Hence, we assume that appropriate models are chosen for different data configurations and parameters and covariance matrix are consistently estimated. Suppose there are *J* hypotheses in total. Let β and $\hat{\beta }$ denote the two *J* × 1 vectors of regression coefficients and their estimates, respectively, one element for each hypothesis. Furthermore, β*_j_* = 0 corresponds to the *j*th null hypothesis, *j*  = 1, …, *J*.

## 2 Multiple Testing Procedures in Clinical Trials

### 2.1 SUM test

O’Brien (2) proposed a test derived from the generalized least squares principle

$$\sqrt{n}{\text{J}}^{\prime }\Sigma^{-1}\hat{\beta },$$

where **J** is an *J* × 1 vector of 1’s and Σ is the covariance matrix of $\hat{\beta }$. When elements of $\hat{\beta }$ are independent from each other, the O’Brien test statistic reduces to a linear combination of ${\hat{\beta }}_{j}$ where each ${\hat{\beta }}_{j}$ is weighted by inverse of its variances. Tests employing linear combinations of ${\hat{\beta }}_{j}$ with different weights have been proposed (3–6), among which the SUM test is especially popular (7). The SUM test statistic has a simple sum form

$$SUM=\sum_{j=1}^{J}{\;}_{j}^{\hat{\beta }}.$$

Under the null hypothesis $\beta_{1}=\cdots =\beta_{j}=0$, *E*(*SUM*) = 0. The SUM test is found to maximize the minimum power (maxmin test) for alternatives where all elements of β have the same sign (8, 9).

### 2.2 Two-step

When homogeneous effects are of interests, a two-step procedure is commonly used. In the first step, we test $H_{0}:\beta_{1}=\beta_{2}=\cdots =\beta_{j}$ versus *H_a_*: at least one pair $\beta_{j}\neq \beta_{j}^{\prime }$ for *j* ≠ *j*′ through Breslow-Day test or likelihood ratio test (LRT) (10, 11). Under the null, the LRT test statistic follows a Chi-square distribution with *J −* 1 degree of freedom asymptotically. If the null hypothesis of homogeneous treatment effects is not rejected, we proceed to the second step where data from different endpoints are pooled and an overall treatment effect is estimated and tested against zero with a Wald test. The second test is carried out conditionally on the acceptance of the null in the first step. When the type I error rates in the two steps, α_1_ and α_2_, both equal 0.05, the marginal probability that the Two-Step procedure concludes homogeneous non-zero treatment effects under *H*_0_ is 95% × 5% = 4.75%, while the probability of concluding non-zero treatment effect in at least one endpoint under *H*_0_ is 95% × 5% + 5% = 9.75%. Lachin and Wei (12) proposed to adjust α_1_ and α_2_ so that the overall type I error rate is α_1_ + α_2_(1 − α_1_) = 0.05.

### 2.3 Approximate likelihood ratio test

The Hotelling’s *T* test examines whether the vector β is a vector of zero

$$n\hat{\beta }\Sigma^{-1}\hat{\beta },$$

Here *n* is the sample size in testing each hypothesis. Under *H*_0_, the Hotelling’s *T* test statistic has an asymptotic Chi-square distribution. Follmann (13) modified Hotelling’s *T* test for one-sided alternatives. His procedure rejects the null when the *p*-value of the Hotelling’s *T* test is less than twice its nominal level and the sum of the treatment effects is in the desired direction (positive or negative). Tang et al. (14) proposed an approximate likelihood ratio test (ALRT) for one-sided alternative hypotheses. A *J* × *J* matrix *A* which satisfies *A*′*A*  = Σ^−1^ and *A*Σ*A*′ = *I* is calculated, where *I* denotes the identity matrix. Define $z=\sqrt{n}A\hat{\beta }$ where the vector $\hat{\beta }$ is mapped into a new vector *z* with independent components *z_j_*, *j*  = 1, …, *J*. For *H_a_*: at least one β*_j_*  > 0, the ALRT statistic is calculated as

$$ALRT=\sum_{j=1}^{J}\max{\left(z_{j},0\right)}^{2},$$

where negative *z_j_* values contribute zero. Hence the absolute magnitude of negative *z_j_* has no impact on ALRT. The ALRT statistic follows a mixed Chi-square distribution under *H*_0_.

### 2.4 MAX test

Another type of global tests employ the maximum of the standardized test statistics (15). The test statistic goes as follows

$$MAX=\max\left(\frac{\left|{\hat{\beta }}_{1}\right|}{SD\left({\hat{\beta }}_{1}\right)},\frac{\left|{\hat{\beta }}_{2}\right|}{SD\left({\hat{\beta }}_{2}\right)},\ldots ,\frac{\left|{\hat{\beta }}_{J}\right|}{SD\left({\hat{\beta }}_{J}\right)}\right),$$

where $SD\left({\hat{\beta }}_{j}\right)$ is the standard deviation of ${\hat{\beta }}_{j}$. Given the one-to-one relationship between $\frac{\left|{\hat{\beta }}_{j}\right|}{SD\left({\hat{\beta }}_{j}\right)}$ and its *p*-value, an equivalent test statistic is the minimum of the *P*-values. The MAX test is powerful to detect alternatives where the treatment effects are non-zero in at least one endpoint/measurement/stratum.

### 2.5 Intersection-union test

Establishment of bioequivalency is required by the U.S. Food and Drug Administration (FDA) in approving generic drugs. The brand-name drug and its generic version are considered indifferent for the *j*th outcome if $\beta_{j}\in \left(-\varepsilon_{j},\varepsilon_{j}\right)$, where the indifferent range ε*_j_* is decided clinically. FDA is interested in whether the generic drug is superior in at least one aspect while non-inferior in all aspects. Therefore, the alternative of interest goes as follows *H_a_*: {max(β_1_, β_2_, …, β*_k_*) > 0} ∩ {min(β_1_+ε_1_, β_2_+ε_2_, …, β*_k_*+ε*_k_*) > 0} where ∩ denotes intersection. The intersection-union test (IUT) (16–18) is most frequently used in these settings. It is a closed procedure which rejects the overall null hypothesis if and only if all null hypotheses included in the procedure are rejected. The *ALRT* is used to test against the alternative *max* (β_1_, β_2_, …, β*_k_*) > 0. Non-inferiority in the *j*th endpoint is tested by

$$\frac{{\hat{\beta }}_{j}+\varepsilon_{j}}{SD\left({\hat{\beta }}_{j}\right)}\;\mathrm{for}\;j=1,\ldots ,J.$$

Because the overall rejection region is the intersection of all rejection regions, the overall type I error will not exceed α if the type I error rates of individual tests are set at α. Although more than one tests are carried out in IUT, it is included in the category of global tests because it draws an overall conclusion, not multiple hypothesis-specific conclusions. The five global tests are summarized in Table 2.

**Table 2:** **Comparison. of five global test statistics**.

Test	Test statistic
SUM	$SUM=\sum_{j=1}^{J}\;{\hat{\beta }}_{j}$
Two-step: step one	$LRT=-2\left(L_{0}-L_{a}\right)$
Two-step: step two	$\hat{\beta }$
ALRT	$ALRT=\sum_{j=1}^{J}\;\max{\left(Z_{j},0\right)}^{2}$
IUT	*ALRT*, $T_{j}=\frac{{\hat{\beta }}_{j}+\varepsilon_{j}}{SD\left({\hat{\beta }}_{j}\right)}$ *j* = 1, …, *J*
MAX	$MAX=\max\left(\frac{{\hat{\beta }}_{1}}{SD\left({\hat{\beta }}_{1}\right)},\frac{{\hat{\beta }}_{2}}{SD\left({\hat{\beta }}_{2}\right)},\ldots ,\frac{{\hat{\beta }}_{J}}{SD\left({\hat{\beta }}_{J}\right)}\right)$

L_0_ and *L_a_* represent the maximum log likelihood under *H_0_* and *H_a_* respectively. And $\hat{\beta }$ is the coefficient estimates from the pooled data.

### 2.6 Comparison of rejection regions of the global tests

**Figure 1:**
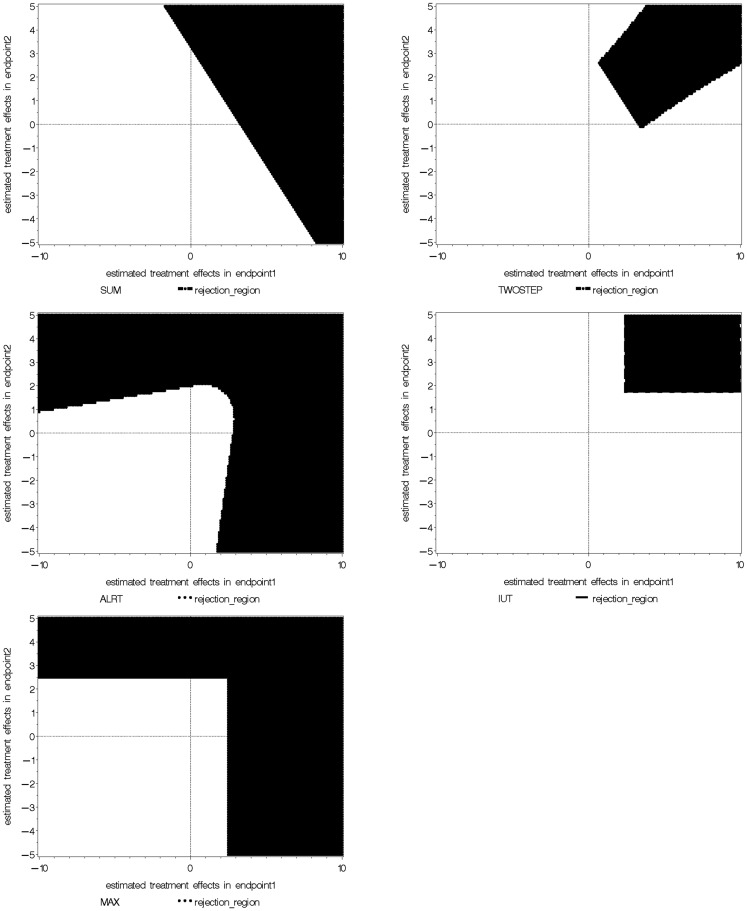
**Comparison of rejection regions of five global tests**.

We take the special example with two coefficient estimates $\left({\hat{\beta }}_{1},{\hat{\beta }}_{2}\right)$, which are are bivariate normal with mean (0, 0), variance 1 and 2 respectively, and correlation coefficient 0.3. The null and alternative hypotheses are *H*_0_: (β_1_ = 0) ∩ (β_2_ = 0) versus *H_a_*: (β_1_ > 0) ∩ (β_2_ > 0). The rejection regions of the five global tests are shown in Figure 1, when α = 0.05 in each individual test. The five rejection regions imply that each test has optimal power against different alternatives. The Wei-Lachin SUM test rejects $\left({\hat{\beta }}_{1},\;{\hat{\beta }}_{2}\right)$ outside a straight line with slope –1 which represents a constant sum. The rejection region of the Two-Step test can be viewed as removing two sides from the rejection region of the SUM test. The MAX test and ALRT reject points with a large positive value in at least one dimension. The rejection region of the IUT eliminates points with negative or close to zero values in any endpoint compared to the rejection region of ALRT.

## 3 Simulation Studies

We simulate binary data following a logistic model to illustrate the global tests. Two different scenarios are examined – correlated multiple outcomes and independent stratified data. For correlated outcomes, each subject *i* has two endpoints. The independent data are from two strata. Two independent covariates are generated: a binomial variable *X*_1_*_ij_* with equal probability to be zero or one and a normal variable *X*_2_*_ij_* with mean 0 and standard deviation 5. The outcomes *Y_ij_* follow Bernoulli distribution specified by *logit*{*p*(*Y_ij_* = 1)} = η*_j_* + β*_j_X*_1_*_ij_* + θ*X*_2_*_ij_*. Note the effects of the treatment *X*_1_*_ij_* is reflected by two endpoint-specific regression coefficients, β_1_ and β_2_. Correlated binary outcomes are generated following Park, Park, and Shin method (19). The intercepts for endpoint 1 and 2 are η_1_ = 0.5, η_2_ = 0.2, and the coefficient for *X*_2_*_ij_* is θ = 0.1 for both endpoints. In simulating the independent binary data, θ = 0.02. In case of unequal sample sizes in the two endpoints, observations in the endpoint with less subjects are missing completely at random. Maximum likelihood estimator for β_1_ and β_2_, as well as the covariance matrix $\sum \left({\hat{\beta }}_{1},\;{\hat{\beta }}_{2}\right)$ are calculated through generalized estimating equations (20). One-sided alternatives H*_a_*: (β_1_ > 0)∩(β_2_ > 0) are tested. Test statistics are calculated using ${\hat{\beta }}_{1}$, ${\hat{\beta }}_{2}$. Each setup is repeated 1000 times. In each iteration, all the test statistics are calculated using the same dataset. We examine and compare the powers and Type I error rates of all five tests for different true values (Table 3), different levels of correlations (Table 4), and different sample sizes at each endpoint (Table 5).

**Table 3:** **Simulation results: size and power (%) with different true value positions**.

True values		ρ	Testing procedure				
β_1_	β_2_		SUM	Two-step	IUT	ALRT	MAX
0	0	0.4	4.9	4.7	0.7	4.4	4.5
0.3	0.3	0.4	21	20	5	18	18
0.6	0.6	0.4	52	50	23	48	46
0.6	0.3	0.4	35	33	11	35	36
0.6	0	0.4	21	17	3	32	33
0.6	−0.3	0.4	11	7	1	33	29

The Type I error 4.7% for Two-Step refers to cases rejected in Step Two. The Type I error for the three tests in IUT are 4.3%, 5.1%, and 4.4%, respectively. The intersection of the three rejection regions gives the overall Type I error rate.

**Table 4:** **Simulation results: power (%) under different correlation between outcomes**.

True values		ρ	Testing procedure				
β_1_	β_2_		SUM	Two-step	IUT	ALRT	MAX
0.6	0.6	0	63	61	18	58	52
0.6	0.6	0.4	52	50	23	48	46
0.6	0.6	0.8	42	40	27	37	41
0.6	0.3	0	44	42	7	40	38
0.6	0.3	0.4	35	33	11	35	36
0.6	0.3	0.8	26	24	12	28	30

ρ Represents the correlation coefficient between the outcomes, not the correlation between the estimated regression coefficient.

**Table 5:** **Simulation results: power (%) with different sample sizes**.

True values		Sample size		ρ	Testing procedure				
β_1_	β_2_	*n*_1_	*n*_2_		SUM	Two-step	IUT	ALRT	MAX
0.6	0.6	100	100	0.4	52	50	23	48	46
0.6	0.6	50	150	0.4	41	37	15	49	30
0.6	0.6	25	175	0.4	30	26	5	51	16
0.6	0.3	100	100	0.4	35	33	11	35	36
0.6	0.3	50	150	0.4	27	26	7	23	21
0.6	0.3	25	175	0.4	18	18	3	23	9
0.6	0.6	100	100	0	63	61	18	58	52
0.6	0.6	50	150	0	55	50	14	55	37
0.6	0.6	25	175	0	36	34	9	54	17
0.6	0.3	100	100	0	44	42	7	40	38
0.6	0.3	150	50	0	37	33	7	47	24
0.6	0.3	175	25	0	24	22	5	48	10

The powers and Type I error rates for different (β_1_, β_2_) values are listed in Table 3. The correlation between Y _i1_ and Y _i2_ is set to be 0.4 and each endpoint has 100 observations. All tests except the IUT maintain the Type I error rates close to the nominal level 0.05. Without prior knowledge of the indifference range, we set the most restrictive indifference range where ε*_j_*  = 0 for every endpoint which is equivalent to requiring all treatment effects to be positive, leading to low overall type I error rate and power. IUT tends to be more conservative than other methods because FDA is more concerned with false positives and only approves new treatment when there is significant evidence supporting its superiority. The procedures can be divided into two groups according to how the power changes when the difference between β_1_ and β_2_ gets larger. The first group includes Wei-Lachin SUM and Two-Step. They are more powerful than the other group when β_1_ = β_2_, but sensitive to non-homogeneous treatment effects. The power of the Wei-Lachin SUM test drops from 52 to 21% when β_2_ drops from 0.6 to 0 while β_1_ remains 0.6. The decreasing trend is even more obvious with the Two-Step. The second group includes ALRT and the MAX test. They are robust to non-homogeneous treatment effects.

In Table 4, 100 correlated pairs (*Y_i_*_1_, *Y_i_*_2_) are generated with various correlation coefficients. All the methods incorporate information from both endpoints. When two outcomes are highly correlated, the treatment effects estimated from both endpoints, ${\hat{\beta }}_{1}$ and ${\hat{\beta }}_{2}$, tend to be similar and provide less information compared to the independent case, hence lower power. However, the IUT has a reversed pattern because with higher positive correlation, the non-inferiority tests on the two endpoints tend to agree more, leading to higher overall rejection rates.

Table 5 lists the different performance of the tests with unequal sample sizes for the two endpoints. When sample sizes are not balanced between the two endpoints, most tests have reduced power because the test statistics combine information from all endpoints and a large variance in one endpoint leads to large variance of the overall test statistic. ALRT is robust to unequal sample sizes. If treatment effects are equal in both endpoints (β_1_ = β_2_ = 0.6), the power of ALRT does not change with the distribution of samples into each two endpoints as long as the total sample size remains the same. When the treatment effects differ in the two endpoint (β_1_ = 0.6, β_2_ = 0.3), the power of ALRT could either increase or decrease depending on which endpoint has more subjects. If the endpoint with a larger treatment effects has a larger sample size, ALRT has higher power. If the endpoint with a smaller treatment effect gets more samples, the power decreases.

## 4 Controlling FWER/FDR in Genomic Studies

### 4.1 Stepwise procedures and FDR

Stepwise procedures are classified into one-step procedures and multi-step procedures. One-step procedures set a uniform threshold for all the unadjusted *P*-values while multi-step procedures set different thresholds for different hypotheses depending on the order of the unadjusted *P*-values. Multi-step procedures can be carried out step-down or step-up (21, 22). In step-down procedures, the hypothesis with the smallest *P*-value is tested first. And as long as one hypothesis fails to be rejected, all the hypotheses with larger unadjusted *P*-values will fail to be rejected. On the contrary, step-up procedures start from the largest unadjusted *P*-value and reject all smaller unadjusted *P*-values after the first one is rejected.

In this manuscript, the FWER is preserved at nominal level in a strong sense, that is, FWER is no larger than the nominal level for testing any subset of the hypotheses set. Given the large number of hypotheses, researchers are often more interested in a more interpretable quantity, the FDR (23). FDR is the rate that the rejected or significant features are truly null. The numbers of true and false positives can be calculated directly from FDR. FDR helps to avoid a flood of false positives when most of the hypotheses are truly null or missing out significant features when the number of true alternative hypotheses is large. FDR can be estimated as

$$FDR=\frac{\pi_{0}mt}{\sum_{j=1}^{J}\;I\left(P_{j}\leq t\right)},$$

where *m* is the total number of hypotheses being tested, π_0_ is the percent of true null among them, *I* is the indicator for a true statement in the bracket, and *t* is the cutoff value of *p*-values to call a feature significant. Although π_0_ is unknown, it can be estimated from the distribution of *P*-values. Benjamini and Hochberg (24) developed a step-up procedure to control FDR at level q*. For ordered unadjusted *P*-values *P*_(1)_, *P*_(2)_, …, *P*_(_*_J_*_)_, we reject the first *j* hypotheses with the smallest *j P*-values if $P_{\left(j\right)}\leq \frac{jq^{*}}{J}$.

### 4.2 Resampling method

Westfall and Young (25) and Troendle (26) developed resampling procedures which simulate the joint distributions of the *P*-values under the null while maintaining their correlation structure. The procedure starts with bootstrap or permutation under the null from the original sample. Then hypothesis-specific pivotal test statistics and the corresponding *P*-values are calculated on the simulated data. The steps are repeated a large number of times to achieve an empirical distribution of (*P*_1_, *P*_2_, …, *P_J_*) under the null which maintains the correlation structure. The unadjusted *P*-values for the *j*th hypothesis is the percent of times the *j*th imputed test statistic is larger than or equal to the *j*th test statistic from the original data. Step-down or step-up procedures can be carried out on the unadjusted *P*-values based on resampling. There is a resampling option in SAS “multtest” procedure for several tests including the two-sample *t*-test, Cochran-Armitage test and Fisher’s exact test. However, this procedure does not allow covariate adjustments and can not be used in multiple comparisons in regressions.

### 4.3 Bonferroni adjustment

The Bonferroni adjustment is a one-step procedure which rejects the *j*th null hypothesis H_0_*_j_* when the *p*-value in testing the *j*th hypothesis $P_{j}\leq \frac{\alpha }{J}$. The FWER in the Bonferroni procedure is conserved at α level because

$$\begin{array}{llll} Pr\left(\text{Reject}\;\text{any}\;H_{0j}|H_{0}\right) & =Pr\left\{\overset{J}{\underset{j=1}{\cup }}\left(P_{j}\leq \frac{\alpha }{J}\right)|H_{0}\right\}\; & & \;\\ & \leq \sum_{j=1}^{J}\;Pr\left(P_{j}\leq \frac{\alpha }{J}|H_{0}\right)\; & & \;\\ & =\sum_{j=1}^{J}\;\frac{\alpha }{J}\; & & \;\\ & =\alpha ,\; & & \; \end{array}$$

where ∪ denotes union. Alternatively, researchers may compute adjusted *P*-values as $P_{j}^{*}=P_{j}\times J$ and compare $P_{j}^{*}$ to the nominal level α. The Bonferroni adjustment is computationally straight forward because the threshold for significant *P*-values in each hypothesis is just the FWER divided by the number of hypotheses. However, Bonferroni procedure is conservative with low power.

Wiens (27) and Huque and Alosh (28) modified the Bonferroni procedure with fixed testing sequence procedure. It allocates the overall Type I error rate sequentially and controls FWER at the nominal level. Let the sequence of hypotheses be $H_{0}^{\left(1\right)},H_{0}^{\left(2\right)},\ldots ,H_{0}^{\left(J\right)}$. Assign type I error rate α*_j_* to each of the null hypothesis such that $\sum_{j=1}^{J}\;\alpha_{j}=\alpha$. Furthermore, if the first hypothesis is not rejected, its portion of the type I error α_1_ will be passed onto the second hypothesis. That is, the type I error rate for the second hypothesis becomes α_1_ + α_2_ conditional on that $H_{0}^{\left(1\right)}$ fails to be rejected. On the contrary, if $H_{0}^{\left(1\right)}$ is rejected, the type I error rate of $H_{0}^{\left(2\right)}$ remains α_2_. In summary, the type I error rates of unrejected hypotheses accumulate and are passed onto the next hypotheses until a hypothesis is rejected or the last hypothesis $H_{0}^{\left(J\right)}$.

### 4.4 Holm, Sidak, and Simes procedures

Holm method is a step-down procedure (29). First, it ranks all the observed *p*-values from smallest to largest *P*_(1)_, *P*_(2)_, …, *P*_(_*_J_*_)_ Compare each P*_j_* to $\frac{\alpha }{J+1-j}$ starting from the smallest *P*_(1)_. Let the first occurrence of $P_{\left(j\right)}>\frac{\alpha }{J+1-j}$ be the *k*th ordered *p*-value. Then hypotheses corresponding to the first *k* *−* 1 *p*-values *P*_(__1__)_, …, *P*_(__k__−1)_ will be rejected and the hypotheses from the *k*th one on corresponding to *P*_(_*_k_*_)_, …, *P*_(_*_J_*_)_ will not be rejected. Alternatively, researchers can also compute the Holm’s adjusted *p*-values and compare them to α. The adjusted *p*-values is based on the ordered *p*-values *P*_(1)_, *P*_(2)_, …, *P*_(_*_J_*_)_ and $P_{\left(j\right)}^{*}=\max_{i\leq j}\left\{\left(J-i+1\right)P_{\left(i\right)}^{*}\wedge 1\right\}$ where ∧ denotes taking the minimum. The adjusted *p*-values are capped at 1 by taking the minimum of $\left(J-i+1\right)P_{\left(i\right)}^{*}$ and 1. Besides, the *j*th adjusted *p*-value is the maximum in the first *j* values, resulting in non-decreasing sequence of adjusted *P*-values.

The Sidak (30) correction assumes that the *J* test statistics are mutually independent and replaces the element-wise *p*-value cutoff α/J by $1-{\left(1-\alpha \right)}^{\frac{1}{J}}$. It is less conservative than the Bonferroni correction because $1-{\left(1-\alpha \right)}^{\frac{1}{J}}\geq \frac{\alpha }{J}$ for *n* ≥ 1. Another set of thresholds combining the Holm threshold and Sidak correction, $1-{\left(1-\alpha \right)}^{\frac{1}{J}},1-{\left(1-\alpha \right)}^{\frac{1}{J-1}},\ldots ,1-{\left(1-\alpha \right)}^{\frac{1}{1}}$, also maintains FWER at α.

Simes (31) procedure is also a step down procedure that rejects *H*_0_*_j_* when $P_{\left(j\right)}\leq \frac{j\alpha }{J}$. Here *P*_(1)_, *P*_(2)_, …, *P*_(_*_J_*_)_ are the ordered *P*-values from smallest to largest. Hochberg and Liberman (32) extended the Simes procedure by allocating different weights to the *P*-values depending on prior information on each hypothesis.

## 5 A Real Case: Genomic Studies Based on a Clinical Trial

We illustrate the stepwise procedures using the Genome Wide Association Study (GWAS) from the Diabetes Control and Complications Trial (DCCT) and Epidemiology of Diabetes Intervention and Complication (EDIC) trial. DCCT and EDIC are two clinical trials based on the same type 1 diabetes cohort in different time periods. The survival rate and life expectancy of type 1 diabetic patients have been improved greatly in recent years. However, chronic hyperglycemia status leads to deleterious changes in blood vessels. Cardiovascular diseases and microvascular complications are major threats to the long-term quality of life of type 1 diabetic patients. This study focuses on microvascular complications among type 1 diabetic patients. In EDIC, 1441 Type 1 diabetic patients enrolled from 1983 to 1989. They were randomized to either the intensive or conventional therapies, where participants in the intensive group monitored and regulated their blood glucose level constantly. DCCT ended in 1993 when significant reduction in the risk of microvascluar complications was found in the intensive therapy group (33). Of the 1441 DCCT participants, 1394 continued to the EDIC trial, where everyone receives the intensive therapy.

The abnormalities in the capillaries lead to symptoms in different parts of the body – nephropathy, retinopathy, and neuropathy. The goal of this analysis is to validate fourteen SNPs associated with severe nephropathy and persistent microalbuminuria in Al-Kateb et al. (34). Urine glomerular filtration rates (GFR), which is an important clinical index of diabetic nephropathy, have been recorded annually in the DCCT/EDIC cohort. Log-transformed GFR values are employed as our main outcome. Linear regressions of the last GFR observation versus each of the fourteen SNPs are fitted, adjusting for age at randomization, gender and duration of diabetes at enrollment, stratified by the treatment group. Different SNP coefficients are assumed in the intensive and conventional treatment groups because patients under the two treatments were in quite different biophysical and metabolic statuses and the intensive control of the glucose level might suppress or activate SNP effects. Among the global tests for SNP effects in different strata, the SUM test is employed as the effects for each SNP are expected to be in the same direction across the two strata and the SUM test is the maxmin test under such conditions.

Fourteen raw *P*-values are generated from the SUM tests, one for each SNP. To maintain the family wise Type I error rate or false discovery rate, four different stepwise procedures are performed – Bonferroni, Sidak, Hochberg, and FDR. All four procedures are directly available in SAS package “multtest.” *P*-values of various procedures are listed in Table 6. We can see that although some raw *P*-values are <0.05, none of the adjusted *P*-values remain significant. That is, after FWER is controlled, the seemingly significant results are not actually significant any more. Among the procedures controlling FWER, the Sidak and Hochberg procedures give smaller adjusted *P*-values and therefore are more powerful than the Bonferroni adjustment. Although researchers usually require the FWER no larger than 0.05, they might set higher cutoff value of FDR depending on the context of the research problem.

**Table 6:** **Real data analysis: association between log(GFR) and 14 SNPs**.

SNP	Minor	Raw	Bonferroni	Sidak	Hochberg	FDR
rs307806	A	0.01071	0.1499	0.1399	0.1499	0.1499
rs2279622	T	0.03383	0.4736	0.3823	0.4398	0.1939
rs4693614	G	0.06319	0.8846	0.5990	0.6924	0.1939
rs11715496	A	0.06702	0.9382	0.6214	0.6924	0.1939
rs8042694	G	0.06924	0.9693	0.6338	0.6924	0.1939
rs2259458	T	0.22639	1.0000	0.9725	0.9791	0.4245
rs3824935	T	0.23555	1.0000	0.9767	0.9791	0.4245
rs2027440	C	0.24256	1.0000	0.9795	0.9791	0.4245
rs2276768	T	0.30994	1.0000	0.9944	0.9791	0.4821
rs10497435	C	0.44626	1.0000	0.9997	0.9791	0.6248
rs3814995	T	0.52058	1.0000	1.0000	0.9791	0.6626
rs2705897	T	0.61445	1.0000	1.0000	0.9791	0.7169
rs7844961	T	0.73593	1.0000	1.0000	0.9791	0.7925
rs4900312	A	0.97914	1.0000	1.0000	0.9791	0.9791

## 6 Discussion

This manuscript reviews methods for the multiple hypothesis testing problem. Five global tests widely used in clinical trials are reviewed: SUM test, Two-Step test, ALRT, IUT, and the MAX Test. The plots of the rejection regions illustrate the different alternatives to which the tests are directed. The SUM and Two-Step tests are powerful for alternatives with homogeneous effects. Two-Step test can be viewed as a modification of the SUM test that incorporates information on how different the treatment effects are and thus more sensitive to non-homogeneous treatment effects. ALRT is robust to not only unequal treatment effects but also unequal sample sizes from the endpoints. MAX test is also robust for non-homogeneous treatment effects. IUT provides information about the overall superiority and individual non-inferiority. In genomic studies, specific conclusions on individual hypotheses are desired and stepwise procedures are commonly used to control FWER or FDR. The Westfall and Young’s resampling method generates the joint distribution of *P*-values under the null and maintains the correlation structure between them. A selected SNP dataset from a clinical trial is used to illustrate the stepwise procedures. Finally, among the hundreds of papers on multiple hypothesis testing topic, only a selected few commonly used multiple hypothesis testing adjustment methods are reviewed here. Our goal is to introduce the classical methods and present the ideas behind them. They serve as the basis on which researchers may choose and develop their own method with careful consideration of the particular research setup and clinical questions.

## Conflict of Interest Statement

The author declares that the research was conducted in the absence of any commercial or financial relationships that could be construed as a potential conflict of interest.
